# A multiplex PCR assay for six Aedini species, including *Aedes albopictus*

**DOI:** 10.1186/s13071-021-04871-7

**Published:** 2021-07-28

**Authors:** Woo Jun Bang, Min Hyeok Won, Seong Tae Cho, Jihun Ryu, Kwang Shik Choi

**Affiliations:** 1 School of Life Sciences, Kyungpook National University, Biology building 226, Daehak-ro 80, Daegu, Korea; 2 Research Institute for Dokdo and Ulleungdo Island, Kyungpook National University, Daegu, Korea; 3 Research Institute for Phylogenomics and Evolution, Kyungpook National University, Daegu, Korea

**Keywords:** Aedini, *Aedes albopictus*, Internal transcribed spacer 2, Multiplex PCR assay

## Abstract

**Background:**

Mosquitoes, as vectors of various human pathogens, are significant drivers of serious human illness. In particular, those species in the Aedini tribe, which typically transmit dengue virus, Chikungunya fever virus, and Zika virus, are increasing their range because of climate change and international commerce. In order to evaluate the risk of disease transmission, accurate mosquito species identification and monitoring are needed. The goal of this work was to develop a rapid and simple molecular diagnostic method for six morphologically similar Aedini species (*Aedes flavopictus*, *Aedes albopictus*, *Ochlerotatus koreicus*, *Ochlerotatus japonicus*, *Ochlerotatus togoi* and *Ochlerotatus hatorii*) in Korea.

**Methods:**

A total of 109 samples were assayed in this study. The internal transcribed spacer 2 (ITS2) regions from all six species were amplified, sequenced and analyzed using Mega 6. Following the identification of regions that were consistently different in terms of sequence between all six species, multiplex primers were designed to amplify these regions to generate species-specific fragments distinguishable by their size.

**Results:**

Uniquely sized fragments were generated in *Ae. flavopictus* (495 bp), *Ae. albopictus* (438 bp), *Oc. koreicus* (361 bp), *Oc. togoi* (283 bp), *Oc. hatorii* (220 bp) and *Oc. japonicus* (160 bp). Pairwise distance analysis showed that the difference was 35.0 ± 1.5% between *Aedes* spp. and *Ochlerotatus* spp., 17.4 ± 0.2% between *Ae. albopictus* and *Ae. flavopictus* and 11.1 ± 0.3% between *Oc. koreicus* and *Oc. japonicus*.

**Conclusions:**

In this study, a multiplex PCR assay for six species of the Aedini tribe was developed. This assay is more accurate than morphological identification and will be useful for monitoring and controlling these vector mosquitoes.

**Graphical Abstract:**

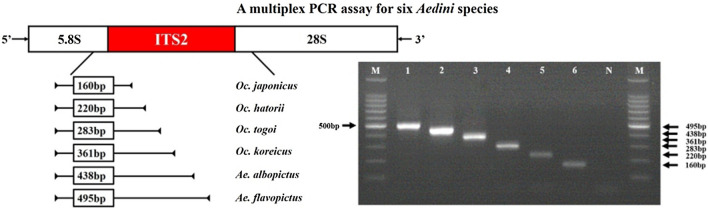

**Supplementary Information:**

The online version contains supplementary material available at 10.1186/s13071-021-04871-7.

## Background

Mosquitoes are vectors of numerous animal and human pathogens, and the threat of mosquito-borne diseases is increasing in parallel with the expansion of the ranges of these mosquito vectors. The expansion of mosquito is driven by climate change and increased introductions into novel territory facilitated by international travel and commerce [1, 2]. More than 250 million people are exposed to mosquito-borne dengue viruses, and more than 400,000 people die from malaria each year [3].

Among the Aedini tribe, *Aedes* spp. and *Ochlerotatus* spp. are known to transmit dengue virus, Chikungunya virus and yellow fever virus, and have been recently shown to be the main vector of Zika virus [4, 5]). The main species that serve as vectors for these diseases are *Aedes aegypti* and *Aedes albopictus*. Notably, the range of *Ae. albopictus*, which is a potential vector for dengue and Zika virus, has been expanding worldwide since the beginning of the twentieth century as climate change and international transport accelerate. It is currently identified as a major invasive species [6, 7]. *Aedes albopictus* is more heat- and stress-tolerant and is more widely distributed in Asia than *Ae. aegypti* [8]. In addition, this species can carry 22 more strains of arboviruses than *Ae. aegypti* and is likely to be a major vector in areas where *Ae. aegypti* is absent [9–11].

In Korea, 12 species of mosquitoes, including *Ae. albopictus*, are known to transmit vector-borne diseases [12]. Every mosquito-borne disease is managed by the Korea Disease Control and Prevention Agency (KDCA) as ‘a legal infectious disease’ necessitating extra surveillance because of the possibility of being introduced from abroad and exacerbated by climate change [13]. Although there have been no reported cases of these viruses as indigenous to Korea, a mosquito infected with dengue virus was found near the airport in Incheon, Korea in 2019, suggesting that the spread of vector-borne diseases due to introductions and climate change is a real threat [14].

Six species of the Aedini tribe (*Ae. albopictus*, *Aedes flavopictus*, *Ochlerotatus japonicus*, *Ochlerotatus koreicus*, *Ochlerotatus togoi* and *Ochlerotatus hatorii*) found in Korea are difficult to identify and distinguish morphologically. *Aedes flavopictus*, a potential vector of the dengue virus, was thought to be restricted to Korea and Japan but has recently been found in Europe [15, 16]. *Ochlerotatus japonicus* has recently spread to North America and Europe and is known to transmit West Nile virus, Chikungunya virus and dengue virus [17–19]. *Ochlerotatus koreicus* is a common species that lives in urban areas, and it is a vector of Japanese encephalitis and Chikungunya virus [20, 21]. *Ochlerotatus togoi*, which is the main vector of lymphatic filariasis, lives in coastal areas of East Asia and also exists along the coast of North America and South America [22–24]. *Ochlerotatus hatorii* is known to be distributed in Korea and Japan [25]. Although the latter species is morphologically similar to the other five species, it has rarely been studied ecologically or biologically.

Successful mitigation of these mosquito-borne diseases requires regular monitoring of the range and population density of their mosquito vectors. This monitoring requires rapid and accurate methods to identify mosquito species. However, morphological identification is difficult, especially if the scales or legs which are used in identification are lost or missing from the specimens [25, 26]. Consequently, the development of a rapid and accurate identification method would be extremely useful. In this study, we describe the development of a new molecular diagnostic method for these six mosquito species using the internal transcribed spacer 2 (ITS2) of the ribosomal RNA (rRNA) region.

## Methods

### Mosquito sample collection and information

**Fig. 1:**
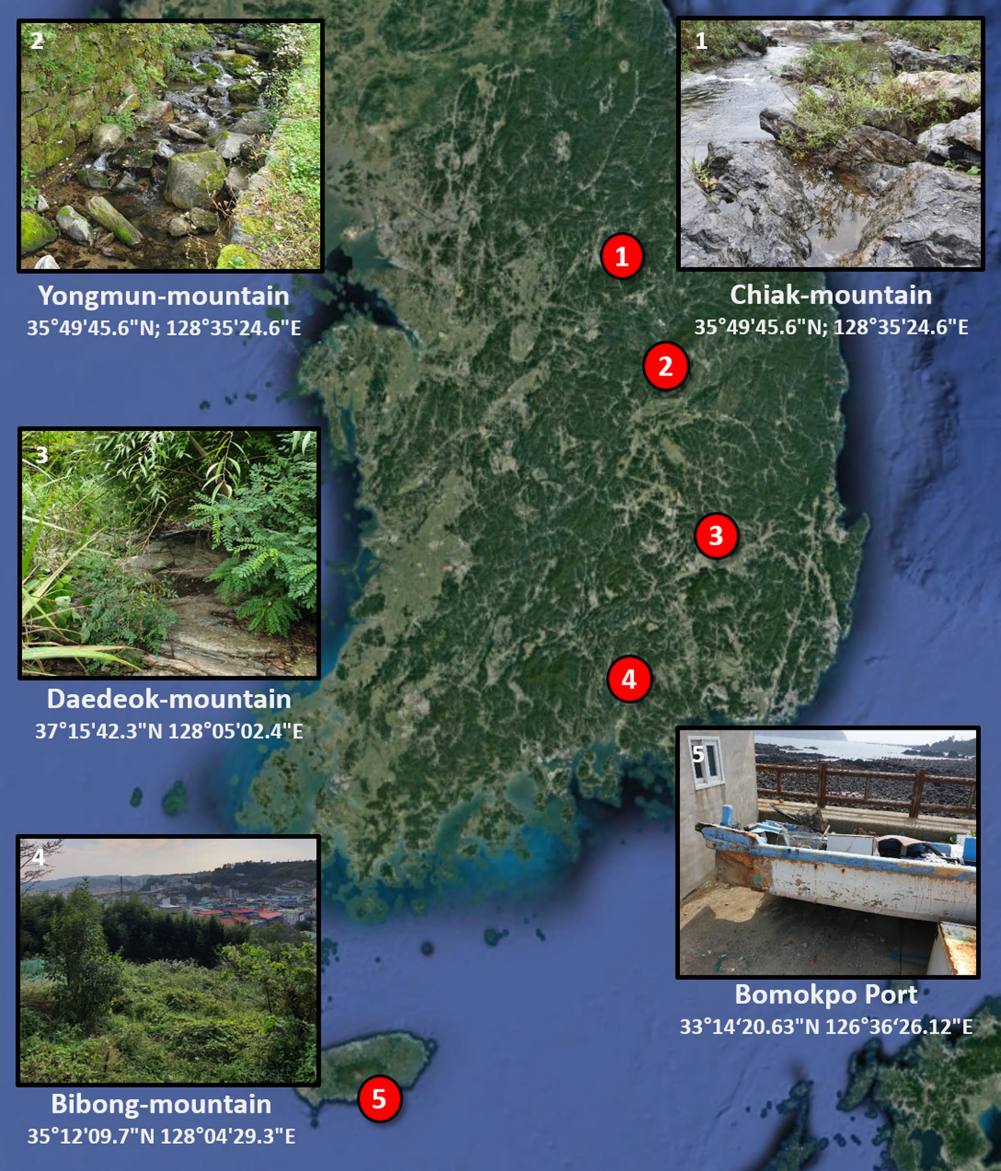
Collection sites of the six Aedini species in Korea. The numbers in red circles indicate the following collection locations:* 1* Chiak mountain,* 2* Yongmun mountain,* 3* Daedeok mountain,* 4* Bibong mountain,* 5* Bomokpo port. The background map image was obtained from Google Earth Pro version 7.3.3.7786 (Accessed 7 Dec 2020)

From August to September 2019, adults and larval mosquitoes were collected from mosquito habitats in five regions of Korea (Chiak mountain, Yongmun mountain, Daedeok mountain, Bibong mountain and Bomokpo port) (Fig. 1). *Aedes. flavopictus* adults (*n* = 20) were collected using BG-sentinel™ (BGS) traps (Biogents, Regensburg, Germany) containing lactic acid and dry ice and then morphologically identified [25, 26]. Larvae of the other five species were collected from water pools around streams and reared to adults in the insectary. Reared adults were identified using known taxonomy keys [25, 26]. Information on the mosquito species and number of specimens collected according to collection site is provided in Table 1.

**Table 1:** Mosquito species and number of specimens collected in each collection site in Korea

Specimens (*n*)	Species	Collection sites					Total (*n*)
		Chiak mountain	Yongmun mountain	Daedeok mountain	Bibong mountain	Bomokpo port	
Collected adults	*Aedes flavopictus*		20				20
Reared adults	*Aedes albopictus*	5		7	12		24
	*Ochlerotatus koreicus*	4		4	12		20
	*Ochlerotatus japonicus*	2		2	1		5
	*Ochlerotatus togoi*					20	20
	*Ochlerotatus hatorii*	20					20

### Sequence determination of the ITS2 region of the six Aedini species

DNA was extracted from each specimen using the AccuPrep® DNA Extraction Kit (K3032; Bioneer Corp., Daejeon, South Korea). Universal primers (forward primer: 5′-AGG ACA CAT GAA CAC CCA CA-3′)/reverse primer: 5′-CTC GCA GCT ACT CAG GGA AT-3′) were designed from sequences registered in GenBank with the following accession numbers: *Ae. flavopictus*—AF353524; *Ae. albopictus—*MN062758; *Oc. koreicus*—KF471622; *Oc. togoi*—EU980394; and *Oc. japonicus*—KF471614. The sequence of the ITS2 region of *Oc. hatorii* was not available when this study was conducted, and so the sequence of the phylogenetically close *Oc. togoi* was used instead [27]. Although four samples for each species were sequenced, one sequence per species was deposited in GenBank due to the absence of within-species variation. The analyzed sequence data were deposited in GenBank under the following accession numbers: MT992619 (*Ae. albopictus*), MW040082 (*Ae. flavopictus*), MW046043 (*Oc. koreicus*), MW046046 (*Oc. japonicus*), MW046045 (*Oc. hatorii*) and MW046044 (*Oc. togoi*).

Each individual reaction mixture (total volume 25 μl) contained 0.4 μM of each primer, 1× PCR buffer, 0.2 mM of each dNTP, 1.0 mM MgCl_2_ and 0.5 U of Taq DNA polymerase (R001AM; TaKaRa Bio Inc., Kusatsu, Shiga, Japan), with 1.0 μl of the genomic DNA extracted from an individual specimen. Amplification was conducted on a Thermal Cycler Dice system (TP350; TaKaRa) as follows: 94 ℃, 5 min; then 94 ℃/30 s, 55 ℃,30 s, 72 ℃/30 s for 35 cycles; with a final extension at 72 ℃ for 10 min. PCR products were visualized in 1.5% (wt/vol) agarose gels stained with ethidium bromide (VWR Life Science, Radnor, PA, USA), and then sequenced in both directions by Macrogen Inc. (Seoul, Korea). Sequences were aligned and analyzed using the Basic Local Alignment Search Tool (BLAST) and Bioedit v7.2.6.1 [28, 29].

### Multiplex PCR assay for the six Aedini species

**Fig. 2:**
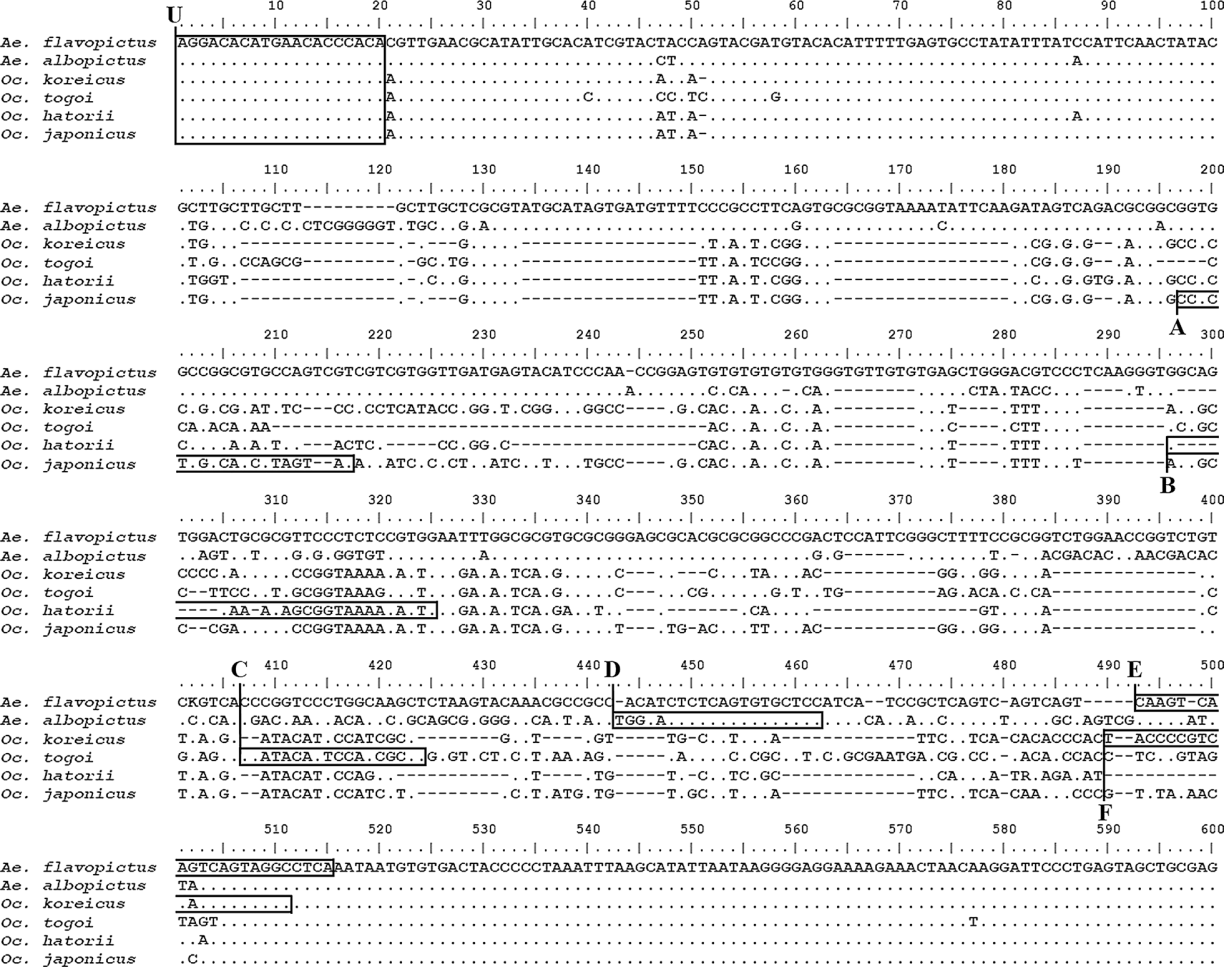
Position of the universal and specific reverse primers within the internal transcribed spacer 2 (ITS2) region.* U* Universal forward primer. Uppercase letters in bold (A–F) indicate *Ochlerotatus japonicus* (A), *Ochlerotatus hatorii* (B), *Ochlerotatus togoi* (C), *Aedes albopictus* (D), *Aedes flavopictus* (E), *Ochlerotatus koreicus* (F)

Six multiplex primer sets consisting of the aforementioned universal forward primer paired with species-specific reverse primers from the ITS2 region were designed (Fig. 2). The multiplex PCR was conducted in a 25-μl reaction volume with 0.4 μM of each primer (Table 2), 1× PCR buffer, 0.2 mM of each dNTP, 1.0 mM MgCl2, 0.5 U of Taq DNA polymerase (R001AM; TaKaRa Bio Inc.) and 1.0 μl of genomic DNA of an individual specimen. The PCR cycling conditions were: 94 ℃, 5 min; then 94 ℃/30 s, 56 ℃/30 s, 72 ℃/30 s for 35 cycles; with a final extension step at 72 ℃ for 5 min. The products were visualized in 2.0% (wt/vol) agarose gels with ethidium bromide (VWR Life Science) and sequenced as described above.

**Table 2:** Universal forward primer sequences and specific reverse primer sequences for the six species of mosquitoes assayed in this study

Species	Forward primer (5′→3′)	Reverse primer (5′→3′)	Product length (bp)
*Aedes flavopictus*	AGGACACATGAACACCCACA	TGAGGCCTACTGACTTGACTTG	495
*Aedes albopictus*		GGAGCACACTGAGAGTTCCA	438
*Ochlerotatus koreicus*		GCCTACTGATTGACGGGGTA	361
*Ochlerotatus togoi*		AGGCGGTGGAGTGTATGG	283
*Ochlerotatus hatorii*		CAATGTTTTACCGCTGTTTGC	220
*Ochlerotatus japonicus*		TATACTACGCTGCCGAGAGG	160

### Phylogenetic analysis of the six Aedini species

The phylogenetic analysis was performed using the neighbor-joining method under the Kimura 2-parameter model. MEGA software version 6 [30] was used to verify phylogenetic relationships and compare these with the results of morphological identification. Bootstrapping based on the ITS2 sequence data was conducted with 1000 replicates, and genetic diversity between the species was compared using pairwise distances.

## Results and discussion

### Comparison of ITS2 sequence and multiplex PCR results

In total, 109 samples of DNA extracted from individual mosquitoes were used for the study (20 *Ae. flavopictus* samples, 24 *Ae. albopictus* samples, 5 *Oc. japonicus* samples, 20 *Oc. koreicus* samples, 20 *Oc. hatorii* samples and 20 *Oc. togoi* samples).

The lengths of the sequenced fragments of the ITS2 regions of the six Aedini species were 580 bp (*Ae. flavopictus*), 576 bp (*Ae. albopictus*), 450 bp (*Oc. koreicus*), 451 bp (*Oc. togoi*), 406 bp (*Oc. hatorii*) and 456 bp (*Oc. japonicus*). The sequence of each fragment was aligned (using BLAST) with the existing sequence registered in GenBank (*Ae. Albopictus*: accession MN062758 Palestine, MF623839, KU497619 China; *Ae. flavopictus*: AF353524, AF353551, AF353553 Japan; *Oc. koreicus*: MK765859 Hungary, JF430391, KF471630 Belgium; *Oc. japonicus*: KF471619 Austria, FJ641870 Belgium, GU121103 USA; *Oc. togoi*: EU980394 Korea) and used to check the binding sites for the universal and specific reverse primers.

**Fig. 3:**
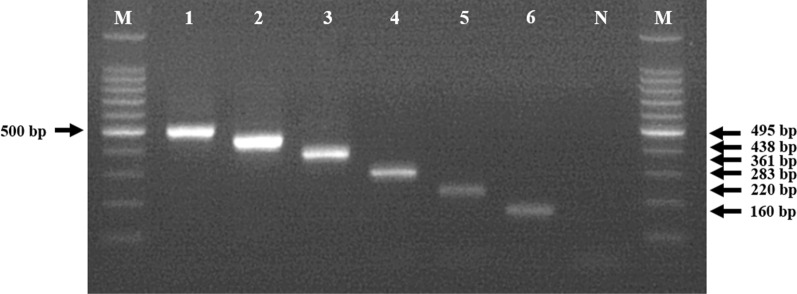
Example of the results of the multiplex PCR assay for six Aedini species. Lanes: *M* 100-bp molecular marker,* 1* *Aedes flavopictus* (495 bp),* 2* *Aedes albopictus* (438 bp),* 3* *Ochlerotatus koreicus* (361 bp);* 4* *Ochlerotatus togoi* (283 bp),* 5* *Ochlerotatus hatorii* (220 bp),* 6* *Ochlerotatus japonicus* (160 bp)

A gel showing the results of the multiplex analysis of DNA from each species is shown in Fig. 3 (*Ae*. *flavopictus* [495 bp], *Ae*. *albopictus* [438 bp], *Oc*. *korecus* [361 bp], *Oc*. *togoi* [283 bp], *Oc*. *hatorii* [220 bp], *Oc*. *japonicus* [160 bp]). The results of molecular analysis were consistent with those of the morphological study.

### Results of the phylogenetic analysis using MEGA 6

**Fig. 4:**
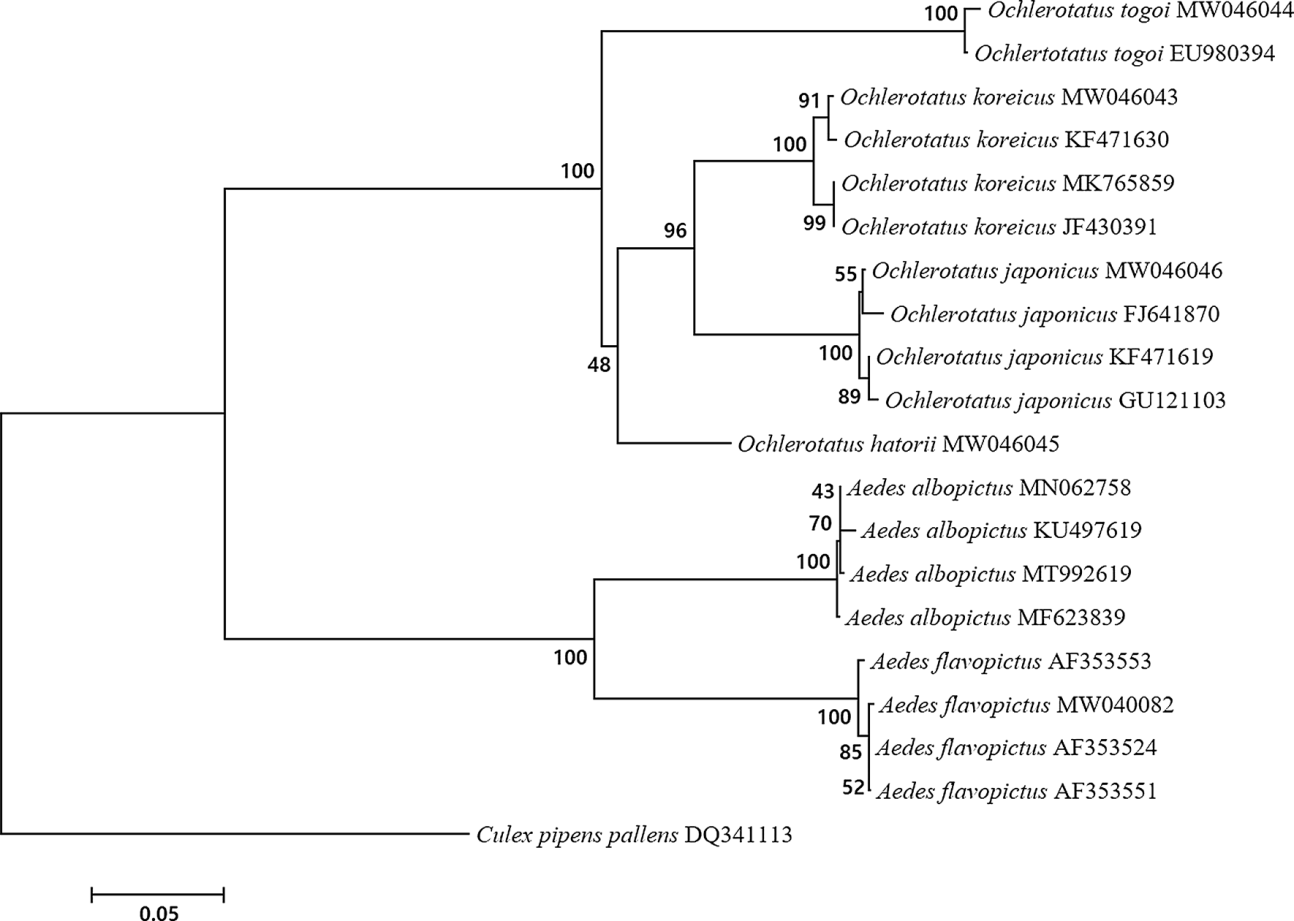
Neighbor-joining phylogenetic tree (Kimura 2-parameter genetic distance calculating method) using ITS2 sequences of six species

The ITS2 results of the six Aedini species sequenced in this study (accessions MT992619, MW040082, MW046043, MW046046, MW046045, MW046044) and other ITS2 sequences deposited in GenBank from other species in the Aedini tribe (accessions KF471630, MK765859, JF430391, FJ641870, KF471619, GU121103, EU980394, MN062758, KU497619, MF623839, AF353524, AF353553, and AF353551) were analyzed to determine phylogenetic relatedness between the six species as well as within the six species (Fig. 4).

*Aedes albopictus* and *Ae. flavopictus*, which are morphologically similar, were more closely related with each other than with the other Aedini species, as were *Oc. koreicus* and *Oc. japonicus*. The phylogenetic tree was clearly divided into *Aedes* spp. and *Ochlerotatus* spp. while *Oc. hatorii* and *Oc. togoi* were only distantly related to the other *Ochlerotatus* spp. A pairwise analysis of genetic distances using the Kimura 2-parameter calculation and the interspecies ITS2 region showed a 35.0 ± 1.5% difference between *Aedes* spp. and *Ochlerotatus* spp., a 17.4 ± 0.2% difference between *Ae. albopictus* and *Ae. flavopictus* and a 11.1 ± 0.3% difference between *Oc. koreicus* and *Oc. japonicus*. The analysis of intra-species variation showed: *Ae. albopictus* 0.44 ± 0.2%, *Ae. flavopictus* 0.41 ± 0.3%, *Oc. koreicus* 1.11 ± 0.7%, *Oc. japonicus* 0.84 ± 0.4% and *Oc. togoi* 0.67% (Additional file 1: Table S1). In addition, the results showed little variation between countries within the species. This is the first report of the results of a phylogenetic analysis of the six species. Although the species are morphologically indistinguishable, the phylogenetic relationship between six species of Aedini tribe as well as between the genera was confirmed by this analysis. There was no discordance between morphological identification and the results of the molecular and phylogenetic analysis for the six Aedini species.

### Application of multiplex PCR molecular diagnostic method

All of the six Aedini species included in this study are very similar morphologically and are identified morphologically by fine differences in the leg, scales, scutum and scutellum [25, 26]. However, the very tediousness of the identification process based on accurate determination of morphological characters and the very real possibility that legs and scales may be lost during collection or storage results in a substantial mis-identification rate. The multiplex PCR assay using the ITS2 region which we developed can reduce this mis-identification rate and is simple—requiring only PCR followed by electrophoresis.

The ITS2 region is located in between the 5.8S and 28S subunits which are conservative with little within-species variation. It is a non-coding region and shows a rapid divergence between species. Also, it is easy to design primers at the conservative regions (5.8S and 28S) and multiple copies with fragments of < 1 kb are present, which is favorable for amplification [31, 32]. Given these advantages, the ITS2 region has been used to identify closely related or morphologically indistinguishable species [33, 34]. A multiplex PCR assay for *Anopheles* spp., which transmit malaria, has also been developed and is being used to monitor certain vector species of *Anopheles* spp. [35–38].

The multiplex PCR assay we developed for Aedini species enables a simple and accurate identification and monitoring of species of mosquitoes that carry *flaviviruses* such as dengue virus, yellow fever virus and Zika virus. Five of the species analyzed here, the exception being *Oc. hatorii*, which is not known to transmit disease, have the ability to transmit pathogens and are currently increasing their respective distribution range. *Aedes albopictus*, which is an endemic species in Asia, has spread into other countries due to the increased trading of waste tires globally [39, 40]. It has also spread to Africa, the Americas and Europe, thereby increasing the probability of infection in these regions [41–43]. *Aedes flavopictus*, which is also an endemic species in East Asia, was first reported in Europe in 2019 [16], and *Oc. japonicus* was reported first in North America in the late 1990s [44, 45] and in Europe in 2002 [46, 47]. *Ochlerotatus koreicus* was recently reported in Belguim in 2008 and in Italy in 2011 [48, 49], and is considered to be a major invasive species together with *Ae. albopictus* in Europe [50]. *Ochlerotatus togoi*, which is endemic species in East Asia and Southeast Asia, was first detected in North America in 1980 [51, 52]. The multiplex PCR assay described here would be a useful tool for monitoring these mosquito vectors in Korea as well as in countries where they were already spread or have the possibility to invade. The data provided by consistent and accurate monitoring of mosquito populations through this method can potentially be used to guide national public health measures, such as quarantine, and thus help prevent the spread of mosquito-borne diseases.

## Conclusions

In this study, a multiplex PCR assay was developed to identify six Aedini species which can transmit various diseases in Korea. This assay provides a simple and accurate molecular identification tool for these six species, which are difficult to identify morphologically. These species are expected to spread globally due to climate change and increasing international trade. This tool will, therefore, be useful for control of the vectors for several infectious diseases.

## Supplementary Information

**Additional file 1: Table S1.** An analysis of pairwise distances using the Kimura 2-parameter calculation for six Aedini species.

## Data Availability

The datasets generated during the current study are included within the article. Sequences used in this study are deposited in the GenBank database under the accession numbers MT992619, MW040082, MW046043, MW046044, MW046045 and MW046046.

## Abbreviations

BLAST: Basic Local Alignment Search Tool
ITS2: Internal transcribed spacer 2
KDCA: Korea Disease Control and Prevention Agency
rRNA: Ribosomal RNA
